# Mentalizing the patient–Patient experiences with short-term mentalization-based therapy for borderline personality disorder: A qualitative study

**DOI:** 10.3389/fpsyt.2022.1088872

**Published:** 2022-12-22

**Authors:** Emilie Hestbæk, Mathilde Hasselby-Andersen, Sophie Juul, Nynne Beier, Sebastian Simonsen

**Affiliations:** ^1^Stolpegaard Psychotherapy Centre, Mental Health Services, Gentofte, Denmark; ^2^Department of Psychology, University of Copenhagen, Copenhagen, Denmark; ^3^Copenhagen Trial Unit, Centre for Clinical Intervention Research, Copenhagen University Hospital – Rigshospitalet, Copenhagen, Denmark

**Keywords:** patient experiences, borderline personality disorder, short-term psychotherapy, mentalization-based therapy, personality pathology, qualitative research

## Abstract

**Background:**

Mentalization-based therapy (MBT) is an evidence-supported psychotherapy approach for borderline personality disorder (BPD) that has been implemented in mental health services worldwide. Originally, MBT was developed as an 18-months program for BPD. However, a short-term (5 months) MBT program has been developed. Research into patient experiences with long-term MBT for BPD is scarce, and no studies have investigated patient experience with short-term MBT for BPD.

**Objective:**

The objective of this study was to explore patient experience with short-term MBT for BPD in the Danish mental health services.

**Methods:**

Semi-structured qualitative interviews were conducted with 12 outpatients diagnosed with BPD, who attended short-term MBT for 5 months. The interviews were verbatim transcribed and analyzed using thematic analysis with double coding.

**Results:**

The analysis resulted in four subordinate themes: (1) *Treatment duration – too short or appropriately short?*, (2) *The group as a “safe space*,” (3) *Bad experiences impacted treatment negatively*, and (4) *My life has changed for the better.*

**Conclusion:**

The results suggest that most of the patients were overall satisfied with short-term MBT, which they experienced as having a positive impact on their lives. However, a subgroup of patients wanted more therapy. This study highlighted the strengths and limitations of short-term MBT for BPD as experienced by the patients, and points to barriers in developing service-user informed short-term treatment options for BPD.

## Introduction

Borderline personality disorder (BPD) is a highly prevalent and severe mental disorder characterized by a pervasive pattern of symptoms such as emotional dysregulation, impulsivity, interpersonal dysfunction, and exceedingly high rates of self-harm and suicide-related mortality (1). Psychotherapy is considered the primary treatment of choice for BPD, and a variety of different interventions exist. According to a recent Cochrane review, both dialectical behavior therapy (DBT) and mentalization-based therapy (MBT) can be considered evidence-supported for treating BPD [Storebø et al. (2)]. Both psychotherapy approaches are significantly superior to treatment as usual on several patient important outcomes, but the certainty of evidence is still considered very low. In addition, the review included a subgroup analysis of duration of treatment, but did not find any association between duration and outcome (2). However, this finding is only indirect and should be interpreted with caution. We are currently performing a systematic review with meta-analysis of short-term versus long-term psychotherapy for adult psychiatric disorders, including BPD (3). As of today, the optimal treatment duration for patients with BPD is currently unclear.

Mentalization-based therapy is a manualized and structured psychotherapy approach, rooted in attachment and psychodynamic theory, that was originally developed specifically for patients with BPD (4, 5). Mentalization refers to the capacity to reflect upon and understand one’s own and other’s mental states, i.e., thoughts, feelings, and desires (4). According to mentalization theory, patients with BPD are more vulnerable to experience frequent and severe impairments in mentalizing, in particularly when emotionally distressed. Impaired mentalization is considered the cause of core difficulties associated with BPD, such as interpersonal- and self-problems, impulsivity, and self-harm. MBT aims at promoting patients’ capacity to mentalize, and to restore it, when lost (4). Importantly, MBT is the only therapy for BPD that has been shown to have long lasting effects on patients after 8-years follow-up (4). Due to the high prevalence of BPD combined with the long-term treatment format, there are often quite long-waiting lists for psychotherapy (6). As a result, MBT is often delivered in different formats and durations for patients with BPD in mental health settings around the world (4). Little is known about the effects of such modifications, including how they are experienced from a first-person perspective by patients.

Recently, a short-term version of MBT has been developed to treat outpatients with BPD (3, 5, 7). The short-term MBT version was developed as a part of a randomized clinical trial assessing the beneficial and harmful effects of short-term versus long-term MBT for outpatients with subthreshold or diagnosed BPD (3, 5, 7). The short-term adaptation is a 20-week program that is overall similar to the original long-term program, but differs in regard to certain parameters: (1) short-term MBT is lower in treatment intensity, i.e., duration (5 versus 14 months), (2) the same therapists provide both individual- and group therapy in short-term MBT (i.e., combined psychotherapy), (3) short-term MBT is structured in closed groups where all participants starts and finish together, and (4) consists of brief psychoeducation (5 weeks) in the beginning, which is delivered in the group. Short-term interventions might be highly useful in resource-limited services, such as public mental health services, due to it being less resource intensive, and its potential to reduce waiting lists and decrease drop-out, which are generally high for patients with BPD (8).

Despite the increasing number of psychotherapy trials for BPD, most research have focused on evaluating the intervention effects of the program in randomized clinical trials, while little attention has been paid to the patient experience of the intervention. However, adding qualitative approaches to randomized clinical trials, has several advantages. For example, the use of qualitative data in combination with quantitative data can strengthen the validity and generalizability of the findings, shed light upon who’s likely to benefit from a given intervention, investigate why a given intervention works for some but not others, and highlight helpful and facilitating factors to improve treatment of BPD (9). Hence, this type of research is of great value both to the patients, relatives, and therapists, and is essential to inform clinical practice and further development and research into short-term interventions for BPD.

Over the past decade, the use of qualitative methods to investigate patient experiences of psychotherapy for BPD is accumulating, with a significant number of studies focusing on DBT (10, 11). To the best of our knowledge, four qualitative papers and one mixed-method paper focusing on patient experiences of MBT, primarily long-term, for BPD has been published (12–16).

Lonargáin et al. (15) explored seven outpatients’ experiences with MBT for BPD (duration ranging from 3 to 14 months). Their main findings were that participants experienced group therapy (open groups) as unpredictable, uncomfortable, and challenging due to difficulties with establishing trust. Indeed, patients highlighted individual therapy as a key ingredient in experiencing positive change (16). In another study, Gardner et al. (14), explored the lived experience of eight patients receiving MBT for BPD (duration ranging from 6 to 9 months) (15). The most salient themes concerned patients experience of their BPD diagnosis and group therapy. Group therapy was described in both negative and positive terms and was regarded as a “necessary evil” facilitating reduction of BPD symptoms. In particularly, the importance of shared experiences and/or learning from being with others was stressed (15). These were also salient themes in a study by Dyson and Brown (13), who explored six patients’ experience of MBT for BPD (duration ranging from 6 to 30 months) (14). In addition, patients’ willingness to change was highlighted as an important aspect of treatment improvement. Even though patients perceived MBT as helpful, the patients did not feel like they were cured (14). In a mixed-method study, Barnicot et al. (12) interviewed 73 outpatients with a personality disorder, primarily BPD, who attended either DBT (duration: 12 months) or MBT (duration: 18 months) (13). The study pointed to helpful and hindering common factors across DBT and MBT for BPD, but also elements unique to the different models. Similarly, Morken et al. (16) explored which therapeutic elements patients with personality disorders, all of whom had significant borderline traits, and comorbid substance use, experienced MBT (duration: up to 3 years), considered useful and less useful (17). Patients stressed the importance of the therapist’s capacity to tolerate strong emotions and address the therapist–patient relationship, as well as being mentalized from multiple perspectives in the group in facilitating improvement.

While previous qualitative studies on MBT mainly include the experience of patients receiving long-term treatment, which cannot be transferred to short-term MBT, there is a gap in the literature. Furthermore, as short-term MBT is currently being used in mental health services worldwide, while also being assessed in a randomized clinical trial, it is important to study the patient perspective. To the best of our knowledge, no such study currently exists. This study seeks to fill this gap by exploring patient experiences with short-term MBT for BPD.

## Materials and methods

The objective of this study was to explore patient experiences with short-term MBT for BPD using a qualitative framework. The Standards for Reporting Qualitative Research (SRQR) has been used to ensure reporting transparency (17).

### Context and clinical setting

This study was conducted in the Mental Health Services of the Capital Region of Denmark. The short-term MBT program was delivered at the Outpatient Clinic for Personality Disorders located at Stolpegaard Psychotherapy Centre. The outpatient clinic specializes in MBT for personality disorders, and all the therapists in this study have received training in the short-term MBT program by national and international MBT specialists, as a part of a large randomized clinical trial (3). The therapists delivering the trial interventions are trained in and provides both short-term and long-term MBT for BPD, i.e., 5- and 14 months.

### The short-term MBT program

Short-term MBT is a 20-week program consisting of five sessions of psychoeducation and introduction to MBT (called MBT-I) followed by 15 sessions of group MBT (MBT-G) in closed groups accompanied by combined individual sessions every second week. Each group consists of seven to nine patients. Furthermore, patients and their relatives are invited to participate in two psycho-educative meetings. Finally, the participants are offered tree individual follow-up sessions after end of treatment. The program is based on the existing MBT manual, which has been modified to the short-term format in close collaboration with the developers (3). The manual is available upon request. See Supplementary Figure 1 for an illustration of the short-term treatment format.

### Design

Semi-structured qualitative interviews were used to collect data about patient experiences with short-term MBT. The interviews were carried out by two research assistants (NB and MH-A). Both research assistants are experienced in conducting structured clinical interviews with patients with BPD, but without prior experience in working with MBT for BPD. Participants were interviewed between 0 and 30 days after finishing their group therapy. The interview guide was designed to capture patient experiences with open-ended questions about the therapy, including structure, format, duration, etc. Nine interviews were conducted face-to-face, while three were conducted *via* telephone due to the COVID-19 pandemic. The interviews that were conducted face-to-face were held in another part of the center apart from the clinic, where they had received treatment. The interviews lasted between 18 and 90 min, with most of them being around 1 h of duration. The interviews were audiotaped and verbatim transcribed. Data were anonymized and pseudonyms were given to the participants.

### Participants, recruitment, and procedures

Participants were recruited using a purpose sampling methodology, that is, all participants enrolled in the short-term MBT program at the outpatient clinic for personality disorders were considered for participation in this study. Clinicians were asked to identify eligible participants, ask if they would be willing to participate, and obtain written informed consent. Participants were included in the study if they complied with the eligibility criteria outlined in Supplementary Table 1. Consenting participants were then contacted by a member of the research team (EH, MH-A) to set up a date for the interview. Recruitment took place from September 2021 to March 2022.

Twelve participants from five different short-term MBT groups were interviewed for this study. This sample size was considered *a priori* as appropriate to conduct in-depth analysis of the individuals’ experiences and to identify and describe themes across participants (18). Participants were on average 26.4 years of age, primarily women (88%), and with Danish nationality (67%). Participants in this study were heterogenous in terms of psychosocial functioning with most of the participants either working (25%) or studying (33%). See Supplementary Table 2 for an overview of patient characteristics.

### Data analysis

The data analysis was conducted using thematic analysis (18). Thematic analysis (TA) requires continuous questioning of assumptions made about data throughout the entire analytical process (18). The identified themes are hereby considered a product of a reflective analytical and subjective process and not something that emerges from the data passively and is simply uncovered by the researchers (18). As an analytical method, TA is theoretically flexible though still constrained by the epistemological assumptions present (18). The epistemological approach chosen for this study was a hermeneutic-phenomenological one, as it entails a focus on the lived experience of the individual in detailed description as well as a continuous process of reflexive circling back in the interpretation of data. TA is useful in making this process overt and concretized to ensure a more transparent approach that is conscious of bias.

To analyze the qualitative data, we applied the methodological principles by Braun and Clarke (18). The analytical method consists of five phases that result in a varying number of themes. *The first phase* entailed the conduction of the interviews, which were performed by MH-A and NB, as well as familiarizing oneself with the data. The interviews were conducted using a semi-structured interview guide and followed by a discussion of each interview between MH-A and NB. To ensure that all parties were familiar with all interviews, MH-A transcribed the interviews performed by NB and vice versa, and EH read through the entirety of the transcribed material several times. *In the second phase*, systematic initial coding of the full data material took place using Nvivo-software (19). Data was dual blind coded by MH-A and NB to keep an open mind toward the data. While coding, notes on the process were made simultaneously as potential themes were beginning to form. *The third phase* involved collecting all codes relevant to a potential theme and collating the extracts. *The fourth phase* involved a refinement of themes through a discussion of the codes that had been formed thus far, in relation to the coded extracts and to what degree the current themes covered the content of them. The relevance of the themes was also considered and revised in relation to the data set as a whole, as dictated by Braun and Clarke (18). Then followed *the fifth phase*, that entailed the process of naming the themes and further defining and refining them. This process involved multiple discussions of each theme between EH, MH-A, and NB continuously circling back to codes and extracts to ensure the themes were representative of the finds. For an overview of the process, see Supplementary Figure 2.

## Results

The analysis resulted in four subordinate themes: (1) *Treatment duration – too short or appropriately short?*, (2) *The group as a “safe space,”* (3) *Bad experiences impacted treatment negatively*, and (4) *My life has changed for the better*.

### Treatment duration – Too short or appropriately short?

Eight participants contributed to this theme by sharing an initial perception of longer treatment duration as inherently better, before they had even started treatment themselves. However, most participants described that looking back now, at the end of their treatment, they had experienced great improvement and now perceived the treatment duration as appropriately short, even though ambivalence toward termination was still expressed. Two subthemes were developed: (a) *In the end, the treatment was appropriately short*, and (b) *I feel like I need more therapy.*

#### In the end, the treatment was appropriately short

One participant reflected upon his view on treatment duration before starting treatment compared to after termination of treatment and how these differed. Before beginning treatment, the participant felt completely overwhelmed by his issues and therefore felt that he would need as much treatment as possible to work through them. However, after terminating treatment, this had changed and he was actually glad, that therapy had not gone on for longer than it did. The participant described it this way:

*“I imagine that if I had been offered treatment for a year, I would have said yes (*…*) because I really needed help, and I had this feeling that there was a lot to catch up on. There is a huge mess inside me, and I want as much [treatment] as I can get. But (*…*) I’m happy that I was only [in treatment] for half a year because it has been helpful, and I was also fed up with it [therapy].”*

This participant also voiced experiencing treatment as long and with periods of time where he had other things going on in his life that he would rather spend time and energy on. This made it difficult and less desirable for him to focus entirely on the treatment during the 5 months. Another participant disclosed a seemingly opposite reaction to being assigned short term-MBT. Looking back, she felt that the duration of treatment had been too short, but at the beginning of treatment the thought of committing to treatment for over a year had seemed overwhelming:

*“It was easier to dedicate myself to six-months of treatment rather than 12-months [ed. 14 months]. That’s why I didn’t choose 12 months, even though I think it could have been more effective when you examine yourself and try to learn different methods of communication with others.”*

Interestingly, one of the participants also described how being assigned the shorter treatment left her feeling like her situation was taken less seriously. However, as stated, she was well satisfied with the treatment in the end.

*“I was worried (*…*) because it was short, and I was scared that not much could happen in half a year (*…*) but I think I’ve been proven wrong. I also think (*…*) that the system has a way of not taking me that seriously. So being assigned the short version instead of the long one was also like, yes, as if maybe it [her situation] wasn’t that bad, but I was actually happy about it [the treatment].”*

Even though most participants seemingly perceived the treatment duration as appropriate, some still expressed ambivalence feelings at the end of the treatment. This included worries about the future, nervousness about being on your own and feelings of sadness related to having to part with the group. One participant described the feelings about terminating treatment as a more general experience as a part of living:

*“But that is also just (*…*) a paradox that you (*…*) experience all the time in your life, That you’re ready, when it’s over. (*…*) So, I don’t know if it can be avoided by the duration of treatment being longer.”*

#### I feel like I need more therapy

Though most participants were satisfied with the duration of treatment, four participants described a wish for more therapy. Of these, three participants explicitly described a history of trauma which they did not feel was possible to work with properly within the timeframe of short-term MBT. These participants pointed to the fact that building trust with the therapists took quite a while and was not fully established until right toward the end. Thus, when the participants finally felt ready to open up and address their traumatic past, the therapy was about to terminate. All four of them described a wish for more individual sessions, and two of them described wanting treatment specifically targeting their trauma. One participant explained it in the following way:

*“In relation to my own personal story I have a very hard time (*…*) trusting other people. And that is a defense mechanism of mine, because throughout the whole of my childhood I have been traumatized and treated horribly.”*

These four participants also shared an emphasis on the wish for more individual sessions more so than expressing a need for prolonging the group sessions. A participant elaborated on this:

*“In the individual therapy sessions, I felt like I compensated for not having the opportunity to talk about the things from my past that I was dealing with that were taking up a lot of space. So, we talked about my past all the time [in individual therapy].”*

Similar viewpoints were expressed by two other participants who had explicitly described a history of trauma. They all shared a positive experience with the treatment which they viewed as helpful but unable to completely accommodate their need for working through what one participant referred to as *“(*…*) a lifetime of issues (*…*),”* especially due to the amount of individual therapy sessions. One out of the twelve participants expressed dissatisfaction with the treatment in general. In particular, she highlighted that an attachment to the group was not able to fully form. She stated:

*“It has probably been a bit short [the treatment]. I don’t really feel like I formed a good enough relationship with the others to be able to share that much.”*

Furthermore, this participant expressed finding most themes of group therapy irrelevant to her situation. She was however satisfied with the individual therapy sessions, which she described as a reason for not dropping out of treatment, even when she found the group sessions irrelevant.

### The group as a “safe space”

Eleven participants contributed to this theme by highlighting the helpful role that closed groups and/or combined therapy played in establishing a safe environment in the therapy. The two subthemes created were: (a) *Closed groups promoted a sense of trust between group members*, and (b) *The individual therapist was an ally in the group and created a sense of coherence in therapy.*

#### Closed groups promoted a sense of trust between group members

Seven participants contributed to this subtheme by describing a positive experience of being in a closed therapy group, i.e., all group members start and finish the group therapy together, and potential dropouts do not get replaced. Most of the participants disclosed that being in a closed group made it feel like a safe space, facilitating openness among the group members and furthering participation in the group sessions. On this matter, one of the participants stated the following:

*“(*…*) I think it was easier to open up. And then you don’t hold back as much. And you get to know each other and feel safe around each other. (*…*) I like that it’s the same people you’re seeing. After all, it’s vulnerable things we talk about. I have previously had much difficulty sharing those sorts of things with other people.”*

Despite an overall agreeance between the participants on a positive experience with being in a closed group, some difficulties were also mentioned. A group member described bringing up a theme concerning her own experiences with sexual assaults, which in her experience was not something that the group could handle. According to her: *“I really felt like, like it was brutal. People were completely silent. (*…*) It seemed like the theme was bigger than us that day*.” Another participant described that she found it difficult to take her place in the group and discuss the themes that were important to her because she felt that they differed from the themes that were relevant to the other group members. Another participant, who also found being in a closed group to be a positive experience, reflected on the possible downsides hereof. She reckoned, that even though being in a closed group was more comfortable and therefore preferable, the exposure to new group members might be beneficial in order to “face your fears.” She elaborated this:

*“(*…*) when you feel anxious about something you need to face it. So, if someone were to come in from outside therapy, then it would be something you could learn to deal with. Because that’s what life is like, and you have to be careful not to be overprotective because of our mental disorder.”*

This reflection was however not shared by the other participants and some even thought of the slow-open group as potentially harmful for the therapeutic process, as it might slow down the process of feeling safe and opening up in the group. One participant even referred to long-term MBT as *“(*…*) the more uncertain long one (*…*)”* which points to the internalized perception that some participants might have of long-term MBT with the slow-open group format.

#### The individual therapist was an ally in the group and created a sense of coherence in therapy

Ten participants stated that conjoined therapy, i.e., the individual therapist being one of the group therapists, was a positive experience in various ways primarily related to establishing trust with therapists and the group, as well as coherence between individual and group therapy. Multiple participants made remarks on the individual therapist being one of the group therapists, which made group sessions feel safer and in turn made sharing more comfortable. One participant specifically mentioned how this affected her desire to share certain things with the group. When asked how she felt about conjoined therapy, she stated:

*“Oh, I thought that was very nice. Because that means that she’s [the therapists] aware of some of the things that the other therapist isn’t (*…*) and it just makes it safer somehow to bring up a topic.*”

Furthermore, several of the participants brought up how the individual therapist was helpful in giving them a gentle “push” at times to be more participating in group sessions. This could be done either by discussing a theme or a situation in an individual session prior to sharing it with the group or by directly encouraging more active participation and supporting the participant when doing so. One of the participants uttered how her therapist would sometimes go about actively encouraging and supporting participation and sharing:

*“She [the therapist] says ‘Okay, what do you think about that?’ (*…*), so that I got to participate a bit more. I thought it was nice that we had talked about some of these things in individual sessions, where she maybe said ‘Come forward’ or ‘Get involved’ or something like that. I felt like she was more aware of me as well, and I found that very nice. (*…*). She did that well.”*

Another participant described how discussing a theme beforehand in an individual session made it possible to share more personal matters with the group. She noted that:

*“(*…*) when there was a conflict in the group, we could use the individual session to find a solution and talk about it. (*…*) I could also pick some more personal things to talk about in individual therapy (*…*) and then I could bring it to group therapy after. I think that worked really really well.”*

The quote also highlights one of the advantages of conjoined therapy, which was described by several of the participants, namely the possibility for discussing potential conflicts in the group therapy in individual therapy sessions. This included a sense of security associated with the individual therapist being in the group to witness potential conflicts and the participant’s dynamics with other group members. This contributed to create coherency between individual and group therapy, which was experienced as meaningful by the participants.

### Bad experiences impacted treatment negatively

This theme was generated from statements of eights participants that revolved around bad experiences in therapy, which included being interrupted, feeling mistrust toward the therapists, and unresolved conflicts with therapists, all of which were not resolved, thereby negatively impacting the treatment going forward.

Though adhering to the timeframe is a significant part of MBT group sessions requiring therapists to interrupt patients and manage the time and format of MBT, four participants experienced these interruptions as highly negative. One participant described how being interrupted and unable to share her point of view with the group as something that made her doubt whether the therapist found her input irrelevant. Another pointed to how it could be tough to be interrupted whilst practicing being vulnerable in group and hoped that there might be a better way to do this. This was supported by a participant who called the interruptions “unbalanced” and something that could leave group members, whose theme had been chosen for that session, looking like they “really” were not feeling well. The interruptions or having to cut off participants while they were talking, seemingly had an impact on the surrounding group members as well, even though they were not the ones being interrupted. One participant elaborated her thoughts on this:

*“(*…*) it was unpleasant on behalf of the others. Because I could see on their faces that they were hurt by it. So, for me, yes, for me it was unpleasant more on their behalf. But you can’t help but wonder if I will be cut off when I try to talk about something that is very important to me?”*

Five out of the nine participants highlighted the impact of conflicts with the therapist and/or therapists on their experience of treatment. Most of the participants that experienced unresolved conflicts with the therapist and/or therapists reported that it influenced the treatment in general. One participant, who had several conflicts with her group therapist during group sessions, stated that she had on multiple occasions considered dropping out due to feeling misunderstood and receiving comments from the group therapist that made her uncomfortable and “not right.” She stated:

*“I mean, I don’t feel like I’ve had any trust towards [the group therapist] at all.”*

Another participant felt that the therapists did not respect her boundaries when they would make her participate in group sessions despite her at times not wanting to. She stated that this created “a lot” of mistrust toward the therapists and made her consider dropping out of treatment. One participant mentioned a certain conflict several times throughout the interview and had experienced it as having multiple negative consequences for her relationship with the group therapist and feeling safe in group therapy sessions. She described a situation where the group therapist interrupted her gesturing a stop-motion with her hand. The participant experienced this interruption as *“(*…*) a slap in the face (*…*)”* and especially the following handling of the situation was perceived as upsetting. She described how she, before the conflict, in that session felt like she was in a good place for the first time since beginning treatment. After the conflict she recounted that:

*“(*…*) Honestly, it was just one small situation. It’s not so much that she did it. The problem is how it was handled afterwards. And that actually made me walk around for a week with anxiety. And it put me back in a situation that was really uncomfortable. So, it overshadowed that I had come really far with many things, which makes this whole situation really severe for me.”*

This conflict seemingly affected in large how the participant perceived her experience of treatment overall, looking back. Furthermore, a participant described an episode that occurred at the second to last group session. The participant had shared an episode with the group and the therapist had made a comment which was described as hurtful.

*“(*…*) I only went to group therapy because I wanted to see the others in the group. I thought being there was a bit unpleasant when she [the group therapist] was there and I was kind of hoping she wouldn’t be there (*…*) And I just thought that it would be a shame if I were to stay away because of that, so I went anyway. So, she apologized to me then. But I still found it difficult.”*

In these situations, the conflicts remained unresolved at the time of termination of treatment and the feeling of therapy being a “safe space” was not restored in time. On the contrary, the breach of trust remained and made the therapeutic situation unpleasant for the participants.

### My life has changed for the better

The theme was created from the statements of eight participants surrounding the discovery of experienced positive changes posttreatment. What seemed to be of importance was especially the discovery and understanding of their own emotional reactions and having achieved better communication skills. This entailed a deeper acceptance and a more positive view on themselves, their emotions, and thoughts. Several participants described how they experienced being more balanced and in control of their emotions, which made them better at handling difficult situations and emotions.

Being in therapy, especially group therapy, was a place where the participants learned to broaden their perspective. Three of the participants highlighted specifically how their mentalizations skills had improved posttherapy and reported that discovering how other people’s minds differ from their own, helped them shift perspective. This was perceived as very helpful for understanding people around them. One patient described that the way the therapist kept repeating questions and focusing on potentially underlying feelings for certain actions or statements was very useful. He described that before starting therapy he would quickly become defensive and aggravated during discussions or disagreements, and that this had changed drastically. He stated:

*“(*…*) Something that has changed is, and it’s only positive, in relation to things like patience towards other people when they are mad at or irritated with me. Or when you’re having a discussion or conflict, I’ve become better at staying calm instead of going off completely. And I definitely think that is what that mentalization-thing has done. Because I have learned to stop and like ‘What is going on here?,’ ‘What is going on with you?,’ ‘What is going on with me?’ Whereas before I had many issues where I was really quick to go off and quickly felt I had to get defensive, you know?”*

This patient described how internalizing some of the therapists’ questions made it possible for him to control impulses, which he had found very challenging beforehand. He also described the change and the impact it had had on his life:

*“I’m much more balanced. And when I wanted to (*…*) pack up everything and leave (*…*): ‘I’m going to go travel for three years,’ ‘I’m going to leave everything behind and say goodbye to everyone.’ Then there’s like a zoom out function where I’m like ‘Is this really what you want?’ and ‘Why am I feeling like this?’ And that is because they [the therapists] kept saying ‘Why do you have this feeling?’ So now I can say to myself ‘But why are you feeling like this right now?’ Maybe it was just because something got cancelled. (*…*) there is just a step between thoughts and action and that’s really really good. I mean, that’s impulse control. So great for my life! That has been a big difference.”*

One patient focused on how being in the group had impacted her social life and interactions outside of therapy. She pointed to the group giving her new positive experiences of sharing thoughts and feelings with the people around her, which in turn seemed to have significant impact on her overall well-being. She stated:

*“It’s one of those things that I will take with me from therapy (*…*) that it’s possible to have this space where you can talk to other people – and it doesn’t have to be other patients. It can be friends from university, it can be family. It can get difficult but, the fact that it is possible is very helpful.”*

Additionally, four participants uttered how their lives were drastically different now from when they first started short-term MBT treatment in terms of level of functioning and quality of life. One participant described it like:

*“I was a completely different person half a year ago. I felt really down (*…*). If I had to talk about something difficult, I would just have to say two words and I would be crying. (*…*) I have become much better at picking myself up and pulling myself together. I’m much happier.”*

In addition to this, another participant described how the positive changes she had experienced posttreatment made her able to “*(*…*) return to life again (*…*).”* One of the participants described how this had improved her quality of life:

*“I feel like communication has gotten easier because I have gotten better at being aware of what is going on. Sometimes. And yes, dealing with conflict, maybe not dealing with conflict, but escalating conflict is not as uncontrolled. I mean I don’t feel like it takes me by surprise that I’m angry (*…*). I feel like you’re not like blind to what’s going on. So, in that respect, it has given me a higher quality of life because I feel that I’ve gotten more control over my actions in daily life.”*

## Discussion

In this study, we explored patient experiences of short-term MBT for BPD in an outpatient clinic for personality disorders in the Mental Health Services of the Capital Region of Denmark. Our analysis resulted in four subordinate themes: (1) *Treatment duration – too short or appropriately short?*, (2) *The group a as “safe space,”* (3) *Bad experiences impacted treatment negatively*, and (4) *My life has changed for the better.* Overall, we found that patients were satisfied with the treatment, which they experienced as having a positive impact on their lives. However, we also found that a subgroup of patients expressed a wish for more therapy. Factors facilitating or hindering improvement were highlighted. This is the first qualitative study to explore patient experiences of short-term MBT for BPD. This study contributes with importance knowledge about how patients with BPD experience therapy and points to hindering and facilitating factors for improvement. The results may also be relevant to other short-term therapy models based on other theoretical models than MBT. Implications and recommendations for clinical practice and future research is discussed.

In regard to the first theme, *Treatment duration – too short or appropriately short?*, many patients described an initial skepticism toward the treatment based on an assumption that “the longer treatment, the better.” Even though this was only evident pre-treatment, this common narrative among the patients may be rather problematic as research show that patients’ expectations and preliminary attitudes to psychotherapy influences the outcome (20). Therefore, it is important that patients perceive their treatment as relevant to them and their situation. In our case, it seems as if much of the skepticism was due to the patients initially not perceiving the treatment as long enough for them to improve. Interestingly, this assumption is somewhat different from the current evidence, which indicates that treatment duration is not related to improvement, that is, long-term treatment for BPD does not seem to be superior to short-term treatment (2). Some participants also associated being allocated to the short-term MBT to a sense of “not being taking seriously” which could be related to both a negative self-image and an epistemic vigilance (1, 21). This may indicate a need for improved dialogue in the preparation phase of short-term treatments. Research stress the importance of pre-treatment preparation and shows that patients who have been prepared thoroughly are more likely to experience higher levels of group cohesion, experience less anxiety, have higher attendance rates, and have more hope in terms of the results of therapy (22, 23). This finding also points to the importance and power of language. In other words, what we name the therapy have connotations and matters to the patients.

Many patients also described ambivalent feelings related to terminating therapy. It was quite common among the patients to describe an increased sense of anxiety about terminating therapy and worries that ending therapy would cause deteriorating of painful emotions. Termination may be particularly difficult for patients with BPD as well as for the therapists working with these patients, as evident in the current study. We have previously proposed a mentalization-based approach to detect and intervene when terminating MBT with BPD patients (24). As an example of a termination-related MBT intervention, we proposed to extend the case formulation with a termination formulation, in which the patient can re-examine treatment goals, addresses how to detect mentalizing failures in the future, specify future mentalizing goals, and attend to unresolved issues with either the other group members or therapists [Juul et al. (24)]. Interestingly, ambivalent feelings related to terminating therapy may not be exclusive to BPD patients. In a recent qualitative study of patients’ experience of transdiagnostic CBT for patients with anxiety disorder and depression (25), also found this. This could indicate that ambivalent feelings at termination may be more of a general phenomenon rather than a diagnosis specific or therapy specific.

As highlighted in the subtheme, *I feel like I needed more therapy*, four patients described a wish for more therapy. Of these, three patients explicitly talked about a history of trauma, and that this was not sufficiently addressed in short-term MBT for BPD. Two patients described how it took them a long time to develop a trustful relationship with their therapists, and that they therefore first started to share and explore sensitive topics, when the individual therapy was about to end. Indeed, all the three patients expressed a wish for more individual sessions. They felt that they could not share more personal issues, either because it was too painful or private, or not appropriate to bring up in the group. According to mentalizing theory, it is assumed that patients with BPD have a disrupted attachment system, which is regarded the root of the core psychopathology, i.e., dysregulated emotion regulation, feelings of abandonment, and interpersonal problems (4). More recently, the MBT literature has focused on the concept of epistemic trust, which refers to ability to trust social knowledge as communicated by others (26). It has been proposed by Fonagy et al. (26) that patients with epistemic mistrust require longer treatment duration as trust and openness first is to be established before patients can begin meaningful psychotherapeutic work. In line with this, Bach and Simonsen (27) suggest that lack of epistemic trust is linked to more severe personality functioning. It is evident that many of the patients with BPD presents with a history of trauma, in particularly childhood trauma (28, 29). More than 30% of patients with BPD also have comorbid PTSD (30, 31), and it is estimated that 40–50% of the patients with BPD also meet the criteria for BPD (32). Based on this, we are strongly encouraging more research into combined BPD and PTSD treatment (33). We hypothesize that a combined treatment format for patients with BPD, who present with trauma, and are distressed by this, may benefit from trauma focused therapy. Thus, patients with more complex trauma, may perhaps require more therapy, but not necessarily longer treatment for BPD. In fact, two of the patients expressed a wish for trauma focused therapy, and not more MBT for BPD. Further, this also highlights the need for more research on epistemic trust, and its role in psychotherapy for psychiatric disorders in general, but in particularly in the case of BPD. Perhaps, offering traumatized patients more individual sessions or separate trauma focused psychotherapy could potentially improve treatment outcome.

In regard to the second theme, *The group as a “safe space*,” patients pointed to the importance of having: (a) the same therapist in both individual- and group therapy, and (b) the closed group format in promoting a sense of coherence and trust. This entailed a subjective feeling of belonging to the group and being supported, which in turn increased patients’ motivation to attend therapy and their willingness to share and explore in the group. These findings are relatively unique to our study compared with findings from previous studies. For example, Lonargáin et al. (15) found that patients experienced MBT group therapy as unpredictable, uncomfortable, and challenging especially due to difficulties with establishing trust, which was exacerbated by the change associated with the open group format (16). Indeed, all participants in their study found the arrival of new members disruptive to therapy, and this was particularly pronounced for the participants who received therapy for less than 5 or 6 months (16). According to the patients in their study, the open-group format interfered with the trust that has already been built among the members, and that the process of trust had to begin all over again with the arrival of a new group members, thus impeding openness in the group (16). Some of the patients in the Lonargáin et al. (15) study also had their individual therapist as one of their group therapists, and similar to our findings, they valued this format, which led them to feel safer and more comfortable in the group. This could correspond to the notion of group cohesion. Group cohesion is regarded as one of the key curative factors in group psychotherapy (20), and is considered the same as therapeutic alliance is in individual therapy (34). Although group cohesion was not directly assessed in this study, patients generally experienced the short-term MBT group as a safe space akin to the concept of group cohesion.

The theme, *Bad experience impacted treatment negatively*, suggests that ruptures in the therapeutic process can impact treatment negatively when experienced as unresolved by the patient. In psychotherapy, rupture, and repair are complex processes that are central to the therapeutic process of MBT and the dynamic within the group and with the group therapist (35). Ruptures can range from tension between group members, between group members and the therapist, disagreements about different elements of therapy, etc. (36). Ruptures can be categorized in two subtypes: one being a confrontational type and the other a withdrawing one (37). When a rupture occurs, it most likely entails a break of trust and misalignment of intentionality that, if repaired, is temporary. In MBT, ruptures create essential opportunities for social development when properly repaired. This entails that a situation of rupture is tolerated and handled by the therapist and that the group members are actively engaged in the process of repair (35). In the short-term MBT program, it is possible that certain aspects of rupture and repair processes differ compared to long-term MBT formats. Certainly, our findings suggest that the reestablishment of epistemic trust in the case of a rupture between a group member and the therapist did not always occur. However, it is uncertain if these ruptures would have been repaired if the MBT group had gone on for longer, or if the repair simply failed without the therapist being aware of the scope of the rupture, as it was experienced by the group members. The process of repair is rarely a straightforward one, which can make complete resolution of a rupture difficult to determine. Gardner et al. (14) found that participants experienced feelings of frustration and rejection due to the timeframe of the group sessions and that they did not seem to be aware of the culprit of these feelings but instead perceived the timeframe negatively (15). Similarly, the participants of the present study did not seem to reflect on why they perceived being interrupted or not having their theme chosen for the group session highly negatively. Awareness of the scope of these frustrations seemingly was not present with the therapists in a way that successfully translated to the participants and their understanding of what purpose the timeframe serves.

Regarding the perceived positive changes following treatment, which was described in the theme, *My life has changed for the better*, one of the main findings of this study was that participants described an increased or new-found ability to take a step back from a situation and be able to assess it differently than before. This included being able to recognize the perspectives of others and how it differs from one’s own view. Morken et al. (16) made similar discoveries with patients, who had been receiving long-term MBT, emphasizing the positive effect of realizing that other’s perspectives could be different than their own and respecting and accepting that (17). Increased self-awareness was another factor that was emphasized as having a positive impact on their life and relationships. In the study of long-term MBT by Lonargáin et al. (15), it was found that the sense of becoming more aware could also include awareness of previous situations where the participants had not been mentalizing and thereby realizing how to better handle situations now, e.g., by not making assumptions about other’s motives (16). This strongly resembles our main findings of participants describing being better at putting themselves in the shoes of other’s including not jumping to conclusions and potentially escalating conflict. This positively affected the patients’ ability to communicate with others, thus leading to improvement in psychosocial functioning. A similar finding was also represented in a study by Gardner et al. (14) which found that the participants experienced improved social skills post-treatment which was linked to improved quality of life and a more positive outlook on life (15). Lonargáin et al. (15) likewise found that participants seemingly had a more positive view on self and others and viewed the future as brighter (16).

### Methodological considerations: Strengths and limitations

This study has several strengths. First, the current study was carried out in the Danish mental health services. Hence, they reflect real world outpatient treatment resulting in high external validity. Secondly, the findings are based on qualitative interviews with 12 patients, which is considered an appropriate sample size for a qualitative study to draw meaningful conclusions (18). Third, the sample was rather heterogenous and represented patients with different socio-demographic backgrounds, ethnicity, and genders. Importantly, the identified themes were clear despite the differences in the samples, thus strengthening the validity of the results.

This study also has some limitations. First, we did not interview any patients, who had dropped out of treatment, and it is likely that they would have provided different accounts of short-term MBT for BPD. Secondly, the interviews were conducted by two research assistants with no prior experience in working with MBT for BPD. This may have resulted in less detailed accounts of the patient experiences due to their limited knowledge and experience with MBT. However, the interviewers lack of clinical experience with MBT could also be considered a potential strength of this study, as it enabled an analytic approach with fewer established assumptions regarding how BPD patients experiences MBT.

## Conclusion

Short-term MBT has recently been developed and implemented in the Danish Mental Health Services. However, research about patient experiences with short-term MBT is lacking. In this study, we found that patients overall were satisfied with short-term MBT, which they experienced as having a positive impact on their lives. However, we also found that a subgroup of patients expressed a need for more therapy. This study contributes with importance knowledge about how patients with BPD experience short-term MBT and points to hindering and facilitating factors for improvement. Implications and recommendations for clinical practice and future research was outlined.

## Data availability statement

The original contributions presented in this study are included in the article/Supplementary material, further inquiries can be directed to the corresponding authors.

## Ethics statement

Ethical review and approval was not required for the study on human participants in accordance with the local legislation and institutional requirements. The patients/participants provided their written informed consent to participate in this study. Written informed consent was obtained from the individual(s) for the publication of any potentially identifiable images or data included in this article.

## Author contributions

EH, MH-A, and SJ: conceptualization and methodology. EH, MH-A, and NB: analysis and data collection. EH: writing—original draft and project administration. EH, MH-A, NB, SJ, and SS: writing—review and editing. EH and SS: supervision. SJ and SS: funding. All authors have read and agreed to the published version of the manuscript.
