# Supplementary material for: Mentalizing the patient–Patient experiences with short-term mentalization-based therapy for borderline personality disorder: A qualitative study
Source: Front Psychiatry. 2022 Dec 22;13:1088872. doi: 10.3389/fpsyt.2022.1088872 (PMC9815536; doi:10.3389/fpsyt.2022.1088872)
Supplement: Supplementary file 1 [file Data_Sheet_1.docx]

**Supplementary material**

**Table 1**

*Eligibility criteria*

| **Inclusion criteria** | **Exclusion criteria** |
| --- | --- |
| - Age 18-60 - Primary diagnosis is emotional unstable personality disorder – borderline type according to the ICD-10 criteria - Completed short-term MBT (max. 20 % absence) - Written informed consent | - Learning disability (IQ < 70) - A diagnosis of schizotypal personality disorder or antisocial personality disorder (DF 60.2) - Another comorbid psychiatric disorder requiring specialist treatment - Current (past 2 months) substance or drug dependence (DF 10) - Concurrent psychotherapeutic treatment outside the clinic - Non-fluent in Danish - Lack of informed consent |

**Table 2**

*Patient characteristics*

| Gender | Age | Primary diagnosis | Ethnicity | Occupation | Level of education |
| --- | --- | --- | --- | --- | --- |
| Woman | 38 | BPD | Danish | Unemployed | Higher Education (≤ 3 years) |
| Woman | 25 | BPD | Non-western | Student | Higher Education (> 3 years)* |
| Woman | 38 | BPD | Other western | Unemployed | Higher Education (> 3 years) |
| Male | 25 | BPD | Danish | Self employed | Higher Education (≤ 3 years) |
| Woman | 22 | BPD | Danish | Employed | Secondary education |
| Woman | 29 | BPD | Non-western | Unemployed | Higher Education (≤ 3 years) |
| Woman | 27 | BPD | Danish | Student | Higher Education (≤ 3 years)* |
| Woman | 19 | BPD | Danish | Student | Secondary education |
| Woman | 22 | BPD | Danish | Unemployed | Primary education |
| Woman | 25 | BPD | Danish | Employed | Secondary education |
| Non-binary | 18 | BPD | Other western | Student | Secondary education |
| Woman | 24 | BPD | Danish | Unemployed | Secondary education |

*Note.* * Currently studying. Abbreviations: BPD = Borderline Personality Disorder.

**Figure 1.**

*Short-term MBT*

**Short-term MBT**

(5 months)

**Case formulation**

(1+1 before MBT-G)

**Relatives are invited twice**

**10**

**individual sessions**

**Group preparation**

**MBT-I**

(5 weeks)

**MBT-G**

**(15 weeks)**

**3 follow-up sessions**

**Figure 2.**

**Data familiarizing**

**Generation of initial codes**

**Identification of themes**

**Revision of themes**

**Definition and naming of themes**
